# Socioeconomic Deprivation as Measured by the Index of Multiple Deprivation and Its Association with Low Sex Hormone Binding Globulin in Women

**DOI:** 10.2174/1874091X01711010001

**Published:** 2017-03-13

**Authors:** Adrian Heald, Ian Laing, David J. McLernon, Rachelle Donn, Andrew J. Hartland, Anthony A. Fryer, Mark Livingston

**Affiliations:** 1 Department of Medicine, Leighton Hospital, Crewe, United Kingdom; 2 School of Medicine and Manchester Academic Health Sciences Centre, University of Manchester, Manchester, United Kingdom; 3 Institute of Applied Health Sciences, University of Aberdeen, Aberdeen, Scotland; 4 Department of Clinical Biochemistry, Keele University School of Medicine, University Hospital of North Staffordshire, Stoke-on-Trent, United Kingdom; 5 Department of Blood Sciences, Walsall Manor Hospital, Walsall, United Kingdom

**Keywords:** Sex hormone binding globulin, Index of multiple deprivation, Type 2 diabetes, Women, BMI, Ethnicity

## Abstract

**Objective:**

Sex hormone binding globulin (SHBG) is a marker of insulin resistance. Given established links between BMI and socioeconomic disadvantage, we investigated how SHBG varies by index of multiple deprivation (IMD).

**Research Design and Methods:**

Using laboratory data from a Midlands UK population of mixed ethnicity, we examined the relation between blood concentrations of SHBG and IMD in 1160 women aged between 17 and 71 years. Women with a serum SHBG >250 nmol/L were excluded.

**Results:**

Mean age was 28.7 (95% confidence interval (CI) 28.2–29.1) years. 48.2% of women were of Caucasian origin, 15.5% of Southern Asian ethnicity and 2.6% were of African or other origin (33.7% were of unknown origin).

SHBG increased with age (Spearman’s ρ=0.195; p<0.001). A higher proportion of women of South Asian origin *versus* other ethnic groups had an SHBG <30 nmol/L (OR 1.93 (95% CI 1.37–2.71)).

SHBG level was lower in individuals with greater socioeconomic disadvantage as measured by IMD (Spearman's ρ= -0.09; p=0.004 for SHBG *versus* IMD).

In multivariate logistic regression, IMD women in the quartiles 2–5 (higher socioeconomic disadvantage) were more likely to have an SHBG <30 nmol/L (compatible with significant insulin resistance) *versus* quartile 1 (odds ratio (OR) 1.71 (95% confidence interval (CI) 1.17–2.53), adjusted for age (OR=0.97 (95% CI 0.95–0.98)) and ethnicity (for South Asian ethnicity OR=2.00 (95% CI 1.42–2.81) *versus* the rest).

**Conclusion:**

Lower SHBG levels in women are associated with a higher level of socioeconomic disadvantage. Given the known association between lower SHBG and higher plasma glucose, our findings suggest a link between socioeconomic disadvantage and future risk of type 2 diabetes.

## INTRODUCTION

Sex hormone binding globulin (SHBG) is the principal transport protein for testosterone and oestradiol. Recent research, however, suggests that SHBG has additional biological significance. Low SHBG concentrations are associated with an increased risk of development of type 2 diabetes mellitus (T2DM) [1]. Low circulating SHBG levels are also associated with insulin resistance [2], itself a characteristic feature of T2DM with insulin resistance playing a major role in its pathogenesis [3].

SHBG is a 90-kDa glycoprotein composed of two 373 amino acid subunits, each with a single steroid binding site. Its principal function has traditionally been considered to be that of a transport protein for sex steroids, regulating circulating concentrations of free (unbound) hormones and their transport to target tissues. SHBG is primarily synthesized in the liver with its production downregulated by insulin [4]. Metabolic clearance from the intravascular compartment is biphasic with the initial clearance occurring over hours and further clearance over a half-life of several days. Glycosylation prolongs serum half-life [5].

Both prospective and cross-sectional studies have reported an association between low serum SHBG concentration and an increased risk of development of T2DM [6]. Of these, 23 cross-sectional and 10 prospective studies were included in a 2006 systematic review and meta-analysis by Ding *et al.* [1] which reported that a higher concentration of SHBG was associated with a lower risk of development of T2DM, and that this relation was stronger for post-menopausal women than for men.

The homeostasis model assessment of insulin resistance (HOMA-IR) using fasting insulin and glucose levels can be used to estimate insulin resistance [7]. Low SHBG has been linked with an elevated HOMA-IR (consistent with increased insulin resistance), in both men and women [4, 8-10].

It is well established that rates of obesity and potentially of insulin resistance are higher in more socially disadvantaged individuals [11-13]. Here, we determined whether index of multiple deprivation (IMD) as a measure of social disadvantage was associated with a lower SHBG in a group of women who had undergone laboratory measurement of SHBG.

Our hypothesis was that greater socioeconomic disadvantage relates to higher BMI and so would be associated with lower SHBG as a marker of insulin resistance in women.

## METHODS

Results of serum SHBG measurements between 1^st^ January 2010 and 31 August 2015 from 1238 individuals were extracted from the department’s laboratory information management system (LIMS) software (Clinisys WinPath v5.32; Clinisys Solutions Ltd, Chertsey, Surrey, UK).

During the study period, all SHBG tests from women were included. Where there was more than one SHBG result at a similar time point the first one was taken. The assay for SHBG was performed on the Abbott Architect immunoassay platform (Abbott Laboratories, Abbott Park, Illinois, USA). Index of multiple deprivation was derived from the full postcode (see below).

Serum SHBG results above the limit of detection of the assay (>250 nmol/L) were excluded (n=78) on the basis that the woman was likely taking an oestrogen containing contraceptive or hormone replacement treatment. This left a final data set of 1,160 women.

An SHBG level of <30 nmol/L was taken as indicative of increased likelihood of impaired glucose regulation [14].

### Indices of Multiple Deprivation

The English Indices of Deprivation combine factors of housing, social and economic issues to give a single deprivation score for small areas (known as Lower-Layer Super Output Areas or LSOAs) in England. An overall weighted aggregation index of multiple deprivation (IMD) is generated based on thirty seven separate indicators, organized across seven distinct domains of deprivation and each area is ranked from the least to most deprived. The criteria and their associated weightings are: Income deprivation, 22.5%, Employment deprivation, 22.5%, Health deprivation and disability 13.5%, Education, skills and training deprivation 13.5%, Barriers to housing and services 9.3%, Crime 9.3% and Living environment deprivation 9.3% [15].

The indices are a widely used standard measure for comparing areas across the country and can help to identify areas with high levels of overall deprivation or areas with specific concerns, health for example, that may not be recognised from the overall index. The measures of deprivation are collected nationally and published every 3-4 years, hence data behind the 2015 IMD are for the year 2012–2013 and as such, will not reflect any policy changes, economic changes or regeneration effects since then. In the IMD 2015, the most deprived LSOA in England is given a rank of 1 and the least deprived is ranked 32,844.

Each SHBG result with an associated postcode was linked to its LSOA using the geoconvert tool [16]. Subsequently, the 2015 indices of multiple deprivation and rank were linked to the LSOA for the specified postcode.

### Statistical Analysis

The data were analysed using MedCalc (version 11.0; MedCalc Software, Belgium). The distribution of continuous variables were assessed using frequency histograms and Kolmogorov-Smirnov normality test. Medians (interquartile range) were presented for skewed data. The Mann-Whitney U-test was used to compare skewed data between the White Europeans and South Asians whilst the Kruskal-Wallis test was used to compare multiple groups (with Dunn’s multiple comparison test when a significance difference was found). Correlations between non-parametric variables and parametric continuous variables were calculated using Spearman and Pearson correlation coefficients, respectively.

A logistic regression analysis was performed to investigate the effect of age, ethnicity and IMD on SHBG as a dependent binary variable. For this analysis, SHBG was coded as 1 for values <30 nmol/L and 0 for values ≥30 nmol/L.

## RESULTS

Data was analysed for 1,160 women. Mean age was 28.7 (95% CI 28.2–29.1) years. The age range was 17–71 years. 48.1% women were Caucasian, 15.2% of women were of South Asian ethnicity and 2.6% of women were of other ethnicity, including African Caribbean or African origin, while in 34.1% of women ethnicity was not known.

Circulating SHBG level increased with age (Spearman’s ρ=0.2; p<0.0001; Fig. (**1**)). For each 5 year increase in age in the women studied, SHBG increased by 16.5%. There was no relation between IMD and age.

By quintile of IMD, SHBG was higher in the most advantaged quintile 1 of IMD (median 46 (interquartile range 29–69 nmol/L)) *versus* quintiles 2–5 (median 39 (26–59)) nmol/L) (H=13.55, p=0.009; Fig. (**2**)).

SHBG was lower in women of South Asian ethnicity (median 33.5 (interquartile range (IQR) 22–53 nmol/L)) *versus* White Europeans (median 42.0 (IQR 28–64); p=0.0001; Fig. (**3**)).

Women of South Asian origin were more likely to have an SHBG of 30 nmol/L or lower, compatible with an elevated risk of dysglycaemia (14) *versus* other ethnic groups (OR 1.93 (95% CI 1.37–2.71)). There was a negative univariate relation between SHBG and random plasma glucose so that a lower SHBG was related to a higher plasma glucose Spearman’s ρ -0.24, p=0.018) for the women on whom both measurements were available (n=97).

There was a negative univariate relation between SHBG and alanine aminotransaminase (ALT) so that a lower SHBG was related to a higher ALT (Spearman’s ρ = -0.30, p<0.0001) for the 282 women on whom both measurements were available, in keeping with the value of SHBG as a marker of hepatic protein synthesis.

### Multivariate Analyses

In multivariate logistic regression, IMD women in the quartiles 2–5 (higher socioeconomic disadvantage) were more likely to have an SHBG <30 nmol/L (compatible with impaired glucose regulation) [14] *versus* quartile 1 (most advantaged) (odds ratio (OR) 1.71 (95% CI: 1.17–2.53), adjusted for age (OR=0.97 (95% CI 0.95–0.98)) and ethnicity (for South Asian ethnicity OR=2.00 (95% CI 1.42–2.81) *versus* other ethnic groups).

## DISCUSSION

We have found that lower SHBG levels in women are associated with a higher level of socioeconomic disadvantage. Given the association between lower SHBG and insulin resistance in both men and women [1, 2, 4, 7, 9, 10] and the relation between low SHBG and higher plasma glucose [14], our findings are further evidence for the link between socioeconomic disadvantage and future risk of impaired glucose regulation.

While the dataset used for our analysis did not include BMI or waist circumference we were able to include a significant number of women across a wide range of IMD with SHBG levels in the analysis.

The fact that the main difference between the quintiles of IMD was between quintile 1, the most advantaged, and quintiles 2–5 of greater disadvantage poses some intriguing questions about differences in lifestyle choices between the most advantaged quintile of the population and the other groups, particularly in relation to exercise levels, calorie intake and efforts to maintain a healthy weight.

As previous studies have shown [17], SHBG increased by age of the woman. This relates to the progressive decline in pancreatic beta cell function with age seen in both women and men [18]. SHBG production at the liver is profoundly downregulated in relation to hepatic insulinisation. With increasing age, hepatic insulinisation diminishes with a resultant increase in hepatic SHBG production with a resultant increase in SHBG as seen here.

We and others have previously shown that SHBG levels are lower in men and women of South Asian origin [14]. The data reported here supported this, with SHBG being significantly lower in women of South Asian origin as a marker of the well-established phenomenon of greater insulin resistance in this group compared with their Caucasian counterparts.

Low SHBG concentration is associated with adverse cardiovascular risk factors [19, 20] and is considered an independent risk marker for the development of T2DM in women [2, 14]. Correlations between low SHBG concentrations and hyper-insulinemia have previously been shown in both health and hyperinsulinemic disease states such as PCOS and T2DM [21, 22]. This strong association has prompted suggestions that a low level of SHBG could be used as a marker to identify individuals with insulin resistance [22, 23].

Weaknesses of the study are the absence of any precise phenotypic data in relation to evidence for PCOS, its cross-sectional nature and the absence of any information about the treatments taken by the women. Nevertheless, the data presented is robust and our finding that IMD is an independent determinant of SHBG level is valid. Any women with very high SHBG suggestive of them taking oestrogen containing preparations were excluded.

Strengths of our study are the number of women included, the diversity of ethnicity of the women with a good number of women of South Asian origin and the wide variation in IMD in the sample.

## CONCLUSION

We have shown that lower SHBG levels in women are associated with a higher level of socioeconomic disadvantage along with ethnic differences between Caucasian *versus* South Asian origin people. Given the known association between lower SHBG and higher plasma glucose, our findings suggest a link between socioeconomic disadvantage and future risk of type 2 diabetes.

## Figures and Tables

**Fig. (1):**
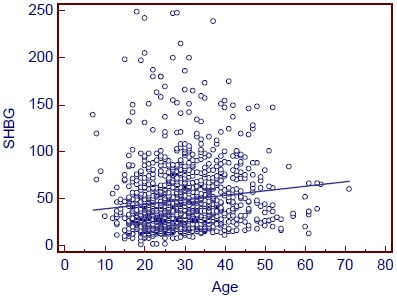
Sex hormone binding globulin (SHBG) level (nmol/L) by age (years) for all women.

**Fig. (2):**
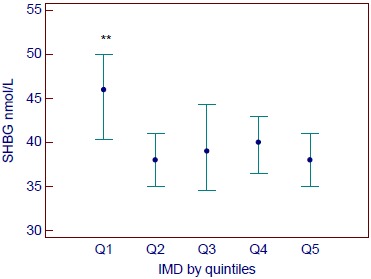
Quintiles of Index of Multiple Deprivation (IMD) *versus* sex hormone binding globulin (SHBG) level (nmol/L). Quintile 1 = lowest deprivation, quintile 5 = highest deprivation. Data are presented as median (interquartile range). **: p=0.009 *versus* quintiles 2–5.

**Fig. (3):**
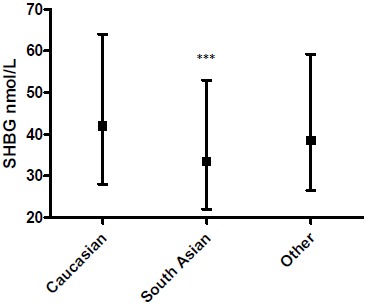
Circulating sex hormone binding globulin (SHBG) level (nmol/L) by ethnic group. ***: p=0.0001 versus Caucasian group.
